# Complementary Methods for the Assessment of the Porosity of Laser Additive-Manufactured Titanium Alloy

**DOI:** 10.3390/ma16196383

**Published:** 2023-09-24

**Authors:** Silviu Mihai Petrișor, Adriana Savin, Mariana Domnica Stanciu, Zdenek Prevorovsky, Marian Soare, František Nový, Rozina Steigmann

**Affiliations:** 1 Department of Technical Science, “Nicolae Balcescu” Land Forces Academy, 3-5 Revolutiei Street, 550170 Sibiu, Romania; silviumihai_petrisor@yahoo.com; 2 Nondestructive Testing Department, National Institute of R&D for Technical Physics, 47 D. Mangeron Blvd., 700050 Iasi, Romania; steigmann@phys-iasi.ro; 3 Department of Mechanical Engineering, Transilvania University of Brașov, 29 Eroilor Blvd, 500036 Brașov, Romania; 4 Department of Impact and Waves in Solids, Institute of Thermomechanics, Dolejskova 5, 18200 Prague, Czech Republic; zp@it.cas.cz; 5 Nuclear NDT Research & Service, 104 Berceni Street, Sector 4, 041919 Bucharest, Romania; soare.marian@nuclear-ndt.ro; 6 Department of Materials Sciences, Faculty of Mechanical Engineering, University of Žilina, 8215/1 Univerzitná, 01026 Žilina, Slovakia; frantisek.novy@fstroj.uniza.sk

**Keywords:** additive manufacturing, titanium alloy, microstructure, ultrasound testing

## Abstract

The method of making parts through additive manufacturing (AM) is becoming more and more widespread due to the possibility of the direct manufacturing of components with complex geometries. However, the technology’s capacity is limited by the appearance of micro-cracks/discontinuities during the layer-by-layer thermal process. The ultrasonic (US) method is often applied to detect and estimate the location and size of discontinuities in the metallic parts obtained by AM as well as to identify local deterioration in structures. The Ti6Al4V (Ti64) alloy prepared by AM needed to acquire a high-quality densification if remarkable mechanical properties were to be pursued. Ultrasonic instruments employ a different type of scanning for the studied samples, resulting in extremely detailed images comparable to X-rays. Automated non-destructive testing with special algorithms is widely used in the industry today. In general, this means that there is a trend towards automation and data sharing in various technological and production sectors, including the use of intelligent systems at the initial stage of production that can exclude defective construction materials, prevent the spread of defective products, and identify the causes of certain instances of damage. Placing the non-destructive testing on a completely new basis will create the possibility for a broader analysis of the primary data and thus will contribute to the improvement of both inspection reliability and consistency of the results. The paper aims to present the C-scan method, using ultrasonic images in amplitude or time-of-flight to emphasize discontinuities of Ti64 samples realized by laser powder-bed fusion (L-PBF) technology. The analysis of US maps offers the possibility of information correlation, mainly as to flaws in certain areas, as well as distribution of a specific flaw in the volume of the sample (flaws and pores). Final users can import C-scan results as ASCII files for further processing and comparison with other methods of analysis (e.g., non-linear elastic wave spectroscopy (NEWS), multi-frequency eddy current, and computer tomography), leading to specific results. The precision of the flight time measurement ensures the possibility of estimating the types of discontinuities, including volumetric ones, offering immediate results of the inspection. In situ monitoring allows the detection, characterization, and prediction of defects, which is suitable for robotics. Detailing the level of discontinuities at a certain location is extremely valuable for making maintenance and management decisions.

## 1. Introduction

The technology of additive manufacturing (AM) and, in particular, metal laser powder-bed fusion (L-PBF) [1,2], also known as selective laser melting (SLM) [3] or selective electron beam melting (S/EBM) [4], has grown from the prototyping phase, today becoming an option for the production of functional or very complex parts due to its multiple advantages, including flexibility in making components, as compared to subtractive manufacturing [5,6]. The realization of complex components (integrating formerly separate parts) by AM benefits from 3D technology in which the model designed in CAD has, as a result, minimal errors [7].

The differences between the morphology of additives-created materials and cast or forged parts cannot be eliminated; in all cases, the requirements imposed by the standards must be ensured and anomalies should be detected and characterized [8]. The parameters of the technological process must be set up (scanning speed, thickness of the layers, chemistry of the surface, etc.), and the powders require certain quality-based microstructural characteristics (grain size, texture, solute, etc.) to ensure that the process does not generate flaws [9].

The production of AM materials offers relatively fast and efficient solutions for complex geometries. Titanium and its alloys, due to their low density, are associated with good mechanical properties, with the high resistance to corrosion required in the manufacture of light parts needed in technological and industrial applications (i.e., aeronautics, the automotive industry, chemical pressure vessels, etc.) [10,11]. The quality and performance of the products obtained by AM by means of the L-PBF method have been intensively studied, establishing that the parameters set in AM for the Ti64 method would have a major impact. In [12,13], the effects of layer thickness, preheating temperature, scanning method, phase structure, and phase transition temperature are studied, and [6] studies the surface morphology. More, reference [14] is focused on the distribution of residual stresses.

The quality of the samples obtained by AM L-PBF is dependent on the properties of the powder used and those of the powder layer [15], as well as on the main parameters in the process (especially the laser’s power and the scanning speed required for a high-quality densification) [3]. Additionally, the conditions related to the cooling rate and the shielding gas [16] can lead to changes in the structure by the latter’s being trapped in the melting pool. Ti64 was part of the first generation of titanium alloys used in medicine. Later, vanadium was found to be toxic to the body, and the cytotoxicity of Al and Fe in the composition and its poor biocompatibility reduced its applicability, limiting it to fields outside of medicine. However, the opportunities to design light structures with complex shapes with minimal cost and essential mechanical properties remained a major attraction for AM for Ti64, especially in the aerospace industry. Obtained in classical shapes of bars, plates, or complex shapes, Ti64 presents good performances, stabilized microstructure, hardness, and good formability.

The progressive deposit of material offers the possibility of quality analysis at each stage, but nevertheless, the combined influence of all parameters can still create process shortcomings [17]. A manufacturing process with deficiencies in set-up can generate mechanical faults in the obtained products. On-site monitoring allows the detection of defects in real time [18]. Thus, the challenge has been moved to quality assurance (QA) and process qualification. Research focused on structural condition monitoring (SHM) and non-destructive evaluation (NDE) for AM applicable to large structures using conventional complementary techniques (e.g., US, X-rays, AE, and ECT) [19,20], can provide potentially relevant data that ensure the profitability of the AM technique and the transition to learning-automated quality control [21]. The framework for this research supposes the existence of an architecture of learning classification on the basis of a dataset obtained from in situ monitoring of AM-obtained components composed of the studied alloy [22].

The classification requires a personalized model of a neural network. Achieving a high degree of quality of simple AM parts and/or components with complex geometries is based on both the design process (data control) and the production process (production and finished-product control). In both cases, it has been found that the selection of materials, 3D printing methods, and measurement methods must be in accordance not only with the specifics and purpose of the product, but also with the economic aspects (when the product requires high precision and durability), as well as the ISO/ASTM standards in force. Obtaining the optimal working parameters for samples through AM L-PBF makes the method vulnerable to process variation, so QA then requires non-destructive evaluation.

According to [19], the indicated method requires continuous monitoring of the process, and also offline non-destructive evaluation by study of the microstructure and physical–mechanical properties. The visual examination of the parts is the first stage of the NDE; it includes the analysis of the continuity of the structure, compliance with the 3D CAD model, and analysis of the state of the texture. Once the conditions for probes are met, knowing how and when different types of defects can appear offers a potential opportunity for intervention in the process. In AM parts, regardless of the method of obtaining them (sintering or fusion based), macro-scale porosity can be present, potentially due to the shielding gas and the lack of fusion [23].

Thermography with short-wave IR allows for the reconstruction of the history of the cause of the formation of the porosity of the part. The presence of defects can also be induced by the equipment, the preparation of the production, or the design of the parts. Being a relatively new method, AM technological processes have recently been improved (the non-destructive evaluation can also include computed tomography (CT) [24,25]), resulting in a limitation of the generation of defects in AM metal. But the costs of the QA equipment and the time allocated require that methods like CT or in situ characterization with synchrotron X-rays be reserved for a limited group of products (e.g., aircraft engine blades) [26]. The components obtained from Ti-6Al-4V (Ti64) by the L-PBF method are usually subject to random and complex mechanical loading. Microscopic flaws such as voids appearing in the matrix, not detected in due time, can develop under conditions of moderate loading and then can be packed, leading to cracks in the part. Applying a QA system, with few economic implications, and following the detection of discontinuities, the probability of detection of defects (POD) increases. The L-PBF process requires an understanding of the thermal mechanisms and physics of fluid dynamics in order to overcome some technical problems related to the formation of defects and changes in the internal morphology. The AM technology facilitates the production of highly complex parts, often piece-by-piece (as in the case of medical implants). Considering this, for QA it has been proved necessary to reevaluate the non-destructive examination techniques applicable to parts manufactured by classical methods. Under these conditions, the selection of complementary non-destructive testing methods to support product QA according to varying thresholds represents a challenge. A complex ultrasonic testing system as a method of investigating the internal structure of AM parts is accepted as a method complementary to non-linear elastic wave spectroscopy or eddy current, ensuring volume testing of the parts. The paper presents the results obtained from the cross-sectional ultrasound examination of the Ti64 samples, parallel to the scanning surface (defined as the C-scan method, according to BS EN 1330-4 2010), being a combination of measurements obtained across the entire thickness of the sample. Ti64 specimens made by L-PBF technology, with internal defects established beforehand, were used in the analysis. The method offers 2D and 3D images of the surface and the inside of the samples, using ultrasonic images in amplitude or time-of-flight. Thus, the discontinuities in the hard-to-reach area are highlighted. Knowing the distribution of defects within the volume of the part offers the possibility of predicting the mechanical properties of AM-manufactured parts. These images, obtained from non-destructive testing, provide information on both QA and compliance with process parameters in the AM.

The paper is structured as follows: Section 2 defines the materials to be tested and the analysis methods (i.e., surface characterization, hardness, and US non-destructive evaluation). Section 3 presents the results of the measurements and their analysis, establishing US C-scan as the method of analysis and characterization of AM samples. Section 4 provides an overview of the results obtained, discussion, and an overview of the possibilities for using US C-scan as a complementary method for inspection of AM samples in aid of further automation.

## 2. Materials and Methods

### 2.1. Materials

**Figure 1:**
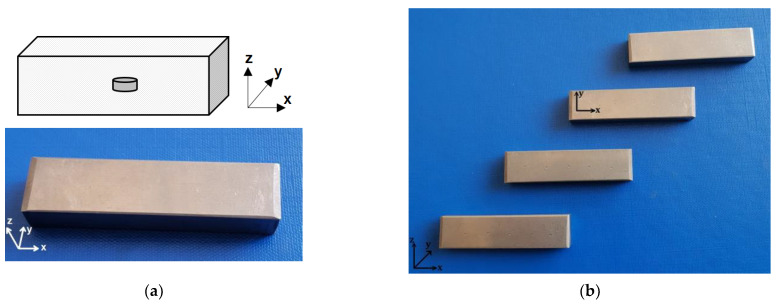
Studied samples: (**a**) photo and schematic of block with inner defects; (**b**)AM samples of Ti64.

Ti64 samples having dimensions of 59.50(X) × 9.40(Y) × 14.49(Z) mm^3^ were prepared by EBM using a process of additive manufacturing on a layer of powder AM L-PBF [27]. The types of samples analyzed are presented in Figure 1. An artificial inner flaw was realized inside each sample; the flaws were of a disk-like shape, with a 5 mm diameter and thickness of 0.2 mm, 0.3 mm, 0.4 mm, or 0.5 mm within an individual sample. The surface of the disk-shaped artificial flaw was parallel with that of the xOy of the sample obtained by AM. The disk-shaped flaws were obtained by modifications during melting. The surfaces have the tendency to sink, deforming the geometry, during cooling. These samples were tested using metallography and by a non-destructive US method.

**Figure 2:**
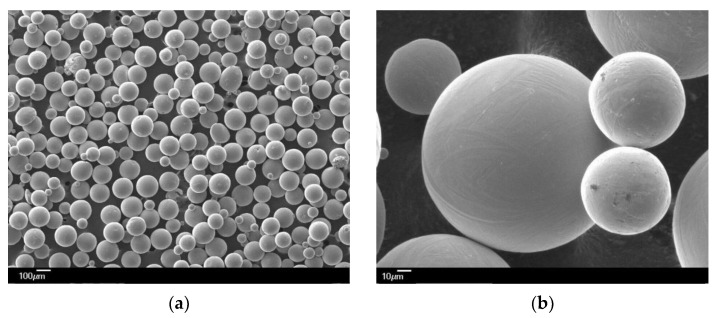
SEMs of Ti64 powder according to [29]: (**a**) X50; (**b**) X500.

The samples have been rendered using spherical powder, grade 5, according to ASTM F2924-14 [28]. The metallic powder Ti64, adaptable to AM [29], has a grain size of 45–105µm, presenting a single-phase Ti64 with a hexagonal compact crystalline structure (hcp) of P63/mmc corresponding to the α phase at low temperatures (in accordance with ICDD—PDF4+:04-002-8708). The SEM analysis of the powder is presented in Figure 2, according to the certificate of analysis [29].

The chemical composition of each sample is presented in Table 1. The values of the tested samples fall within the ranges of values defined by ASTM B348-09. The fine powder was chosen to avoid the potential occurrence of granulation which leads to excessive porosity in the final production of the samples.

### 2.2. Experimental Methods

#### 2.2.1. Surface Characterization

Optical evaluations were carried out on the surfaces of the samples, and the final composition was analyzed for each sample. The tests were accomplished according to ASTM 1476-04, using a SPECTRO xSORT spectrometer. The prediction of porosity, which strongly affects the mechanical performance of a product, becomes of major importance [30], and it depends on the pre-processing parameters [31], the prediction of the model being sensitive to local changes even under the conditions of training on labeled data. The SLM samples were obtained using the Arcam Q20 + system Concept Laser M2 PBF machine (General Electric, Gothenburg, Sweden), and were then processed directly, without introducing additional stress. To analyze the microstructure of the surfaces, a chemical attack with a mixture of HF (20%), HNO_3_ (10%), and water H_2_O (70%) was carried out. The samples thus prepared were investigated using a Zeiss AXIO Observer Z1 m (Zeiss Group, Jena, Germany) metallographic microscope.

#### 2.2.2. Brinell Hardness Test

The micro-hardness tests were performed on the samples in accordance with “EN–ISO 6506–1:2015” at 5 randomly chosen points, using the Nemesis 9102-Innovatest (INNOVATEST, Maastricht, The Netherlands) equipment.

#### 2.2.3. The Non-Destructive Evaluation Based on Ultrasound (US)

The samples obtained by AM L-PBF considered in the study have as induced defects flaws related to processing—microstructural defects. A void can be considered to be a nanoscale group, the concentration of which can increase with temperature. In [32] it was found that a low density of voids (<1% vol.) does not affect the cyclic or tensile testing. However, it cannot be ignored that a pair of voids can work together to initiate a stress fracture. The non-destructive evaluation based on US has been widely applied in the characterization of conventional materials as well as advanced ones. Although it is mentioned in the AM literature, it is not mentioned as frequently as one would expect in the case of classical production. The US technique is applicable in the case of AM for the detection of porosity [33], assessing the quality of finished parts, and the detection of flaws and the determination of the microstructure.

Usually, the C-scan ultrasonic non-destructive method consists of using a container full of liquid (usually water) and an arm that assures a certain position in four axes (x, y, z, and angle θ) for the emission–reception transducer. Dedicated software on a computer assures control of the system and displays the C-scan results. US testing is carried out according to “ISO/ASTM TR 52905:2023 Additive manufacturing of metals—Non-destructive testing and evaluation—Defect detection in parts”.

**Figure 3:**
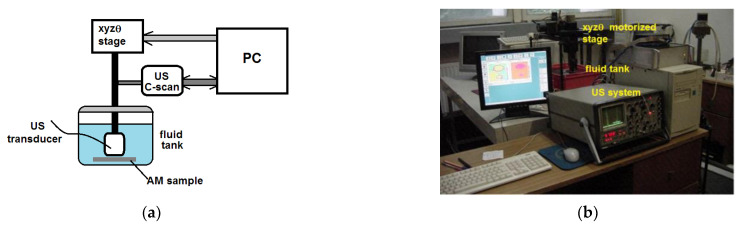
Ultrasonic equipment: (**a**) schematic diagram; (**b**) MicroSonic—Mark IV device.

The detection and evaluation of the simulated defects help with the parameter calibration and set-up of the NDE. For the samples described above in Table 1, the applied strategy for US methods consists of the use of the Nuclear MicroSonic—Mark IV ultrasonic equipment of the Nuclear NDT Research&Service Ltd., in accordance with its quality management system. The US equipment is represented in Figure 3. The equipment consists of a fluid tank, a controller of the mechanical displacement, (X, Y, Z, θ), the US emission–reception system, a data acquisition board, and an industrial workstation.

The software for the equipment can display the data in 2D format, allowing the analysis of the A-, B-, and C-scan images to identify defects or other discontinuities. During the US testing of samples #1 to #4, C-scan images were identified as the best method for viewing the expected results. The C-scan US method is often used to highlight cracks, corrosion, or inclusions/porosity in the parts. Using this method, the mapping of inclusions/defects in the samples is obtained by projecting the US data on a plan view of the tested area, thus displaying an image.

**Figure 4:**
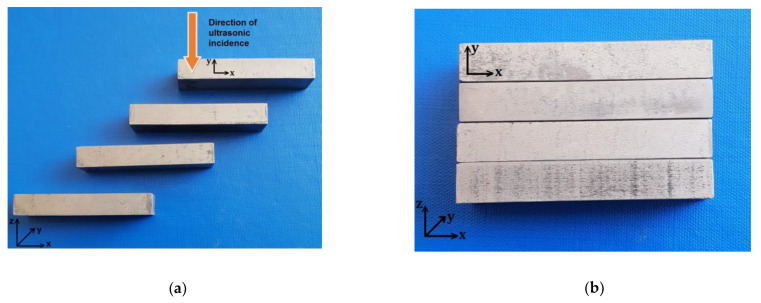
Studied samples: (**a**) direction of testing; (**b**) sample layout for testing.

The X–Y scan was performed, using a resolution of 0.3 mm in both directions. Small dimensional defects inside of the samples were emphasized during the US examination. For scanning, the samples were placed next to each other, with the lengths parallel to the X axis, the incidence of the ultrasonic normal beam being the one indicated in Figure 4a (exposed surface). The transducer swept the surface of the samples placed next to each other (Figure 4b), with the length parallel to the X axis. The methods used were time-of-flight as well as normal beam back echo.

## 3. Results

The AM LPBF method for obtaining the samples is sensitive to variation of process parameters; by evaluating the microstructure, it can be observed whether the samples are dense and if the microstructure is adequate. The microscopic analysis can offer a confirmation that the process parameters were maintained the same for all samples.

### 3.1. Surface Characterization

**Figure 5:**
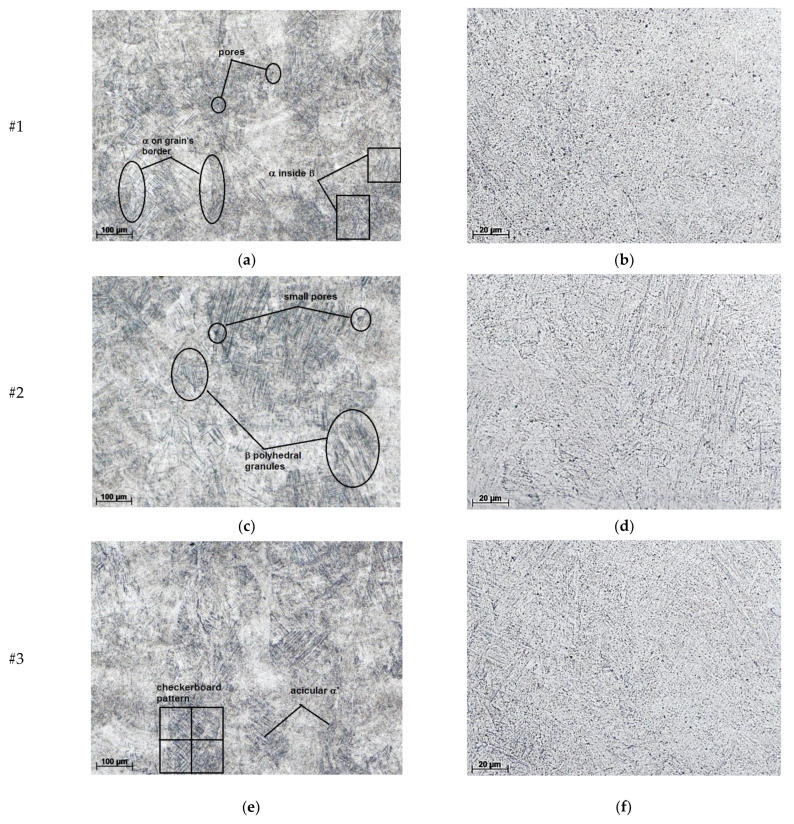

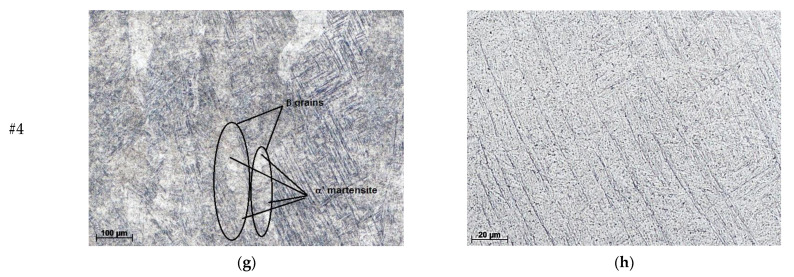
Metallography of samples studied: (**a**,**c**,**e**,**g**) 100× magnification; (**b**,**d**,**f**,**h**) 500× magnification.

Figure 5 presents the microscopic analysis after the samples were polished after metallographic attack. The images emphasize a structure with fine-grained lamellae, dispersed particles of α phase in the grain’s borders, and lamellae of the α phase inside the β-phase grains.

Figure 5a,b show images characteristic of SLM sample structures produced under the conditions of establishing the optimal parameters for the material’s surface (good densification and without major flaws), without large pores and surface cracks; this sample is designated sample #1. This situation can be observed in all samples taken in the study, samples #2 and #3 presenting a stronger texturing.

Figure 5c–f show microscopic images of the sample surface in which the presence of several smaller pores (less than 30 µm in size) can be seen, and in which polyhedral granules with lengths between 150 and 300 µm can be seen. These are primary grains in the β phase due to the exceeding of the deposition temperature of Ti64 [3,34].

Directional solidification and repetition of thermal cycles in the SLM technique can result in an unbalanced microstructure with columnar β grains. The material obtained by AM LPBF presents a mixture of α (light-colored) and β (darker-colored) phases, the two phases being spread in a texture. Partial remelting of previously solidified layers can lead to a strong β solidification texture [35,36].

As for sample #1, it is possible that there was a lack of fusion detection due to insufficient penetration at the high scanning speed. Besides scanning speed, laser power combined with layer thickness can also lead to defects such as lack of fusion. However, the possibility is not excluded that the shielding gas was trapped in the melting pool due to rapid cooling [37].

Samples #2 and #4 (Figure 5g,h) show an almost perfectly dense microstructure under the same scanning conditions. For sample #3a, original β grains in the shape of a checkerboard and acicular α’ martensite from the inside can be observed, as reported by [38]; contrastingly, in #4, the original β grains are arrayed in columns parallel to the direction of construction and acicular alpha prim martensite can be detected on the inside [32].

### 3.2. Hardness Results

The results of the Brinell hardness tests on the samples obtained by AM LPBF are presented in Table 2. It can be seen that the tests, effectuated in several points, have closely-associated hardness values despite the small differences of microstructure.

### 3.3. Defect Detection and Characterization

Obtaining the optimal working parameters of the samples through AM L-PBF makes the method vulnerable to process variation, so QA requires non-destructive evaluation. According to [20], the method requires continuous monitoring of t(us)he process, and also offline NDE by study of the microstructure and physical–mechanical properties. The US method is one of the most common non-destructive testing methods for all materials and is based on the acquisition of ultrasonic waves with frequencies between 1–15 MHz.

The US speed is a parameter that can detect small changes (about 0.5%) in the total porosity. The US pulse–echo method is highlighted as being effective for testing the physical–mechanical characteristics of AM parts at greater depths than those offered by X-rays and is comparable to neutron diffraction; it does not compare with them, though, in terms of resolution. The application of the C-scan technique requires a focused transducer with high sensitivity for the detection of pores with dimensions below 1 mm. Metallography analysis of Ti64 has shown low porosity. Under these conditions, in order to cause sufficient attenuation variation, it was necessary to use an ultrasonic transducer with a smaller beam focal diameter to have a higher sensitivity with high threshold levels.

The ultrasonic C-scan images were obtained during the test, and a focused-point transducer of 15 MHz was used with an effective diameter of 0.25” and a focal-distance length of 2”. As a coupling medium, fine mineral oil was used to prevent any possible corrosion phenomenon, specifically, superficial oxidation. Ultrasonic dimensions and depths of flaws, as determined from ultrasonic imaging, are given in Table 3. The impulse–echo method was used for determinations of longitudinal wave propagation velocity, with a transducer with 5 MHZ central frequency G5KBGE placed on a delay block and connected with a Pulser PR5077 (Olympus USA). The value for ultrasonic velocity has been obtained from the average time-of-flight and average thickness measured in each sample. There were not significant differences between the samples. The longitudinal wave US velocity was determined to be 4380 m/s and this value has been used for the calculation of the flaw’s depth.

Table 3 presents the interval between the minimum and maximum values of an ultrasonically detected flaw’s depth, obtained from determination of time-of-flight corresponding to the flaw’s echoes (ultrasound reflections from the flaw), and with a speed of ultrasound of 4380 m/s. These differences are due to the fact that the surfaces of flaws are not perfect; thus, from the US reflections on this surface, multiple echoes are obtained, all with different times-of-flight.

There are significant differences with the diameters of the intended defect (5 mm). Thus, the differences are greater as the thickness of the defects decreases (from sample #4 to sample #1).

In the conventional impulse–echo method, the depth of the flaw is found out by comparing the amplitude of the signal reflected from the flaw with that from reference notch. However, since the reflected signal from the flaw is adversely affected by flaw’s orientation, surface roughness, transparency, etc., this technique tends to underestimate the size of the real flaw. The time-of-flight (TOF)-based techniques are based on monitoring the time-of-travel of the diffracted signal from the flaw’s top surface. Since the TOF is dependent on the flaw depth alone, the sizing accuracy achieved by this technique is excellent. The TOF-based depth-sizing technique can be used during normal-beam as well as angle-beam pitch–catch scans.

The ultrasonic time-of-flight (US TOF) method is a common technique in the field of non-destructive testing (NDT) of materials; the technique is based on a signal transmitted from an ultrasonic transducer and received at a later time by the same or a different transducer. From the time delay between transmitting and receiving the signal, properties such as the speed of sound or the distance travelled can be derived. In ranging applications, the main objective is to localize distant scatters in front of the transmitter.

**Figure 6:**
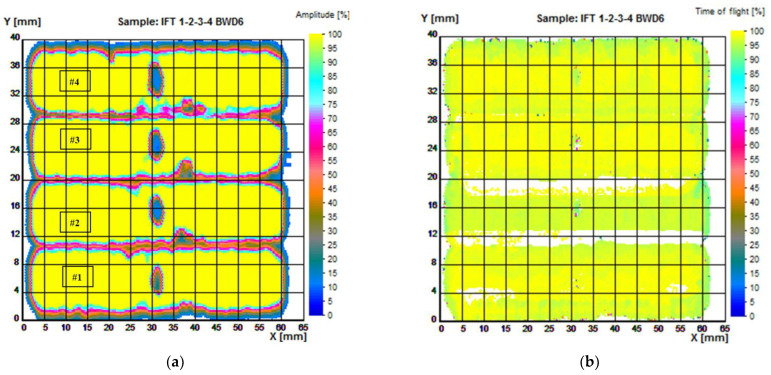
Ultrasonic testing of samples 1 to 4: (**a**) amplitude image, examination ISA-FE5 on back echo; (**b**) time-of-flight image, examination ISA-FE5 of back echo.

The C-scan back-wall echo images of the AM samples are presented in Figure 6. Ultrasonic C-scan displays data along with depth (time-of-flight) or wave amplitude. The percentages of the amplitude and time-of-flight results are derived from the ratio between the individual values measured from an acquisition point and the maximum values allowed by the equipment on the respective channels. Depth information can be obtained by using time-of-flight methods, as indicated by different color shadings.

Amplitude differences were analyzed for AM samples (#1 to #4) with internal defects induced during processing. It can be seen from the amplitudes of the signals, the data being presented at high resolution, that the defects are placed at the same depth below the surface. This technique also gives a good estimate of the dimensions of the defects in the circumferential–axial plane.

Knowing how and when different types of defects are likely to occur offers a potential opportunity for intervention in the AM process. In these parts, regardless of the method used to obtain them, macro-scale porosity can be present, having as causes the shielding gas and the lack of fusion [16]. The presence of defects can also be induced by equipment, the preparation of the realization, or the design of the parts.

**Figure 7:**
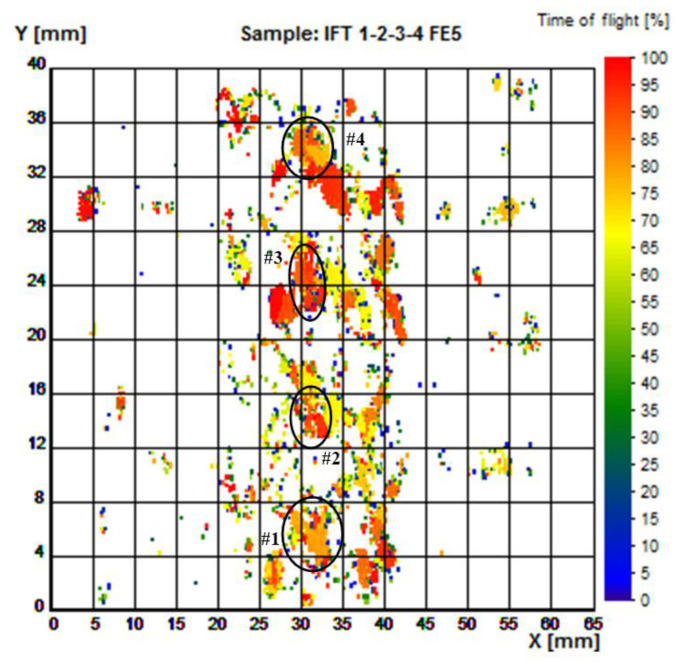
Time-of-flight image of samples 1 to 4, examination ISA-FE5, as to flaw echo. Used for determination of flaw depth in respect to the surface of incidence.

Figure 6 is based on the amplitude and time-of-flight (TOF) of the back echo, and Figure 7 is based on TOF of the flaw echo.

Figure 7 emphasizes the spatial distribution of the flaws induced during the obtaining of the part, and several inhomogeneities of internal microstructure which do not appear as amplitude results in Figure 6.

In the case of the flaw echo, the dependence of the spectral properties on the diameter of the defect is more complex, since the effect of backscattering the acoustic energy (which determines the main echo, selected on the gate) overlaps with the effect of mode conversion at the surface of the defect (which determines the secondary echo, which does not intervene) in the spectral analysis (but it selectively “absorbs” from the spectral content of the incident beam), and both are strongly frequency-dependent phenomena. However, it is important to note that the spectral domains of the two signals (back echo and flaw echo, respectively) are distinct: the high-frequency area for the flaw echo and, respectively, the low-frequency area for the back echo in the presence of the defect. Obviously, the spectral window (the “gap”) between the two frequency domains is all the greater as the diameter of the defects is limited to a smaller value, in relation to the section of the incident ultrasonic beam.

High frequencies are in the middle of the beam, in the region of −6 dB. The reflections from the flaws, which form the flaw echo, eliminate high frequencies if the flaw has a diameter close to the diameter of the beam at −6 dB. Indeed, in this situation, the spectral difference between flaw echo and back echo is maximal. If the flaw has a smaller diameter than the diameter of the beam at −6 dB, then, after reflection, a much higher frequency would pass and the difference between the two echoes would decrease.

The analysis of the US map offers the possibility of information correlation, mainly as to flaws from certain areas, as well as to the distribution of specific flaws in the volume of the part (herein: flaws and pores). Final users can import C-scan results as ASCII files in order to carry out deep analysis with advanced algorithms. Knowing the spatial distribution of flaw types in the part’s volume favors the prediction of mechanical properties of AM-manufactured pieces. The methods based on the measurement of the propagation time evaluate the velocities of the different elastic waves that appear in the material as well as their attenuation.

The results presented using the ultrasonic C-scan method on Ti64 AM show that the determinations can be performed in a relatively optimal time interval, being complementary to other non-destructive testing methods, and can complete other methods of testing AM-processed parts. This proves to be an important application for inspecting the defects inside AM-manufactured samples at a high gain. In the first stage of this method, several dense samples with different flaws defined in advance have been tested. The characteristics of the flaws have been observed (they had changed positions inside the sample) and the circular shape of the flaws couldn’t be determined. The ultrasonic method is one of the most precise methods for determination of the shapes, dimensions, and depths of volumetric discontinuities. The errors/uncertainties are determined, in particular, by possible inhomogeneities of acoustic–elastic properties leading to variations of propagation velocities and of reflection coefficients/reflectivity values of discontinuities.

## 4. Conclusions

The paper demonstrates the potential of C-scan ultrasound to emphasize the internal flaws of Ti64 parts made through L-PBF AM; only those with thicknesses of 200 µm, 300 µm, 400 µm, and 500 µm have been investigated, as realized in hard-to-reach areas.

The AM technology allows the induction of artificial flaws into a piece in order to improve the ability of NDT methods to detect it. The investigation was accompanied by the analysis of the final composition of each sample and of its microstructure. A good densification and surfaces without defects were observed, which denote the obtaining of AM samples under the conditions of having chosen the optimal parameters. The hardness tests performed at several points on the surfaces of the parts show a structure with small pores, with measurements below 30 µm. A structure made up of fine-grained lamellae, the dispersed particles of the α phase on the grain boundaries, and α-phase lamellae inside the β-phase grains were highlighted. At one stage, the presence/absence of flaws can be emphasized in the images, and a report can be generated for further use of the data in machine learning. Combining the signals processed by the different pieces of equipment that assure nondestructive evaluation, the monitoring of the additive-manufactured products might be carried out. The ultrasonic imagistic analysis might be a precise and flexible method for the characterization of AM products. The possible errors and uncertainties are within relatively small ranges for AM products that are not too complex, and for which in situ monitoring is feasible, either by immersion in fluids or by sonic coupling by fluid jet. Although these tests depend on the geometry of the sample (only parallelepiped samples were tested), the method could also be applied to samples with complex geometries that have a large curvature radius, collecting big data.

Future works will consist in the development of other complementary methods such as multifrequency eddy current and non-linear elastic-wave spectroscopy for nondestructive testing of AM complex parts with flaws in hard-to-reach zones.

## Figures and Tables

**Table 1:** Ti64 chemical analysis results (wt.%).

Sample		Al		V		Fe	Ru, Cr, Ni, Cu, Nb, Zr, Sn, Pb	Ti
		Trans	Long	Trans	Long			
#1		5.78	5.40	4.26	4.30	0.26	<0.02	Balance
#2		6.76	4.96	4.22	4.32	0.24		
#3		6.98	4.83	4.22	4.33	0.23		
#4		5.59	5.27	4.34	4.24	0.25		
Titanium Grade 5 B348-09	min	5.50		3.50		-	-	-
	max	6.75		4.50		0.40	-	-
Uncertainty		±0.27		±0.12		±0.05	-	-

**Table 2:** Brinell microhardness of Ti64.

Sample	HB2.5/187.5					Average Value HB2.5/187.5
	1	2	3	4	5	
#1	320	314	313	322	319	318
#2	313	313	316	312	320	315
#3	316	314	313	312	314	314
#4	318	315	319	316	315	317

**Table 3:** US results.

Sample	Sample Dimensions [mm]	Ultrasonic Diameters of Flaws [mm × mm]	Ultrasonic Flaws Depth [mm]
#1	59.42 × 9.40 × 14.47	2.8 × 3.6	8.1 ÷ 8.3
#2	59.50 × 9.34 × 14.38	3.2 × 4.0	8.6 ÷ 9.0
#3	59.34 × 9.31 × 14.47	3.2 × 3.8	8.6 ÷ 9.0
#4	59.37 × 9.28 × 14.49	3.6 × 4.8	8.1 ÷ 8.6

## Data Availability

Not applicable.
